# Improving Function in Older Veterans with Low Vision Using New Accessible Technology

**DOI:** 10.1093/geroni/igaf122.3793

**Published:** 2025-12-31

**Authors:** Sonia Madera, Gabino Lares, Tahira Tyrell, Mythili Bharadwaj, Alyssa Benjamin, Nora Carolina Cabrera, Ivana Yero

**Affiliations:** Veterans Administration, Fort Lauderdale, Florida, United States; WPB VA MEDICAL CENTER, West Palm BEach, Florida, United States; WPB Veterans Administration, West Palm BEach, Florida, United States; Veterans Administration, West Palm BEach, Florida, United States; VETERANS ADMINISTRATION, WEST PALM BEACH, Florida, United States; VETERANS ADMINISTRATION, WEST PALM BEACH, Florida, United States; VETERANS ADMINISTRATION, WEST PALM BEACH, Florida, United States

## Abstract

**Introduction:**

Vision decreases with aging. In the US approximately 18% of people over 65 demonstrate some form of low vision leading to a major impact on ADLs and quality of life with increasing dependence and potential institutionalization. We have demonstrated objectively using FIM scores that accessible technologies such as ORCAM can have a significant impact on improving the ADLs of older adults with low vision. A major drawback is the prohibitive cost of $4500 for the device. Recent advances have adapted the common iPhone to utilize similar programs which are applied on smart devices (Android & iOS) at no cost to the user.

**Objectives:**

Comparing the IPHONE and ORCAM in improving functional independence in Veteran older than 60 to regain their desired level of independence using apps on smart devices with training provided.

**Methodology:**

comparing data from the Functional Independence Measurement (FIM) scale in comparing the ORCAM and mobile phone app (Seeing AI).

**Results:**

Since 2015, 210 Veterans were trained on mobile phones. Technology can capture images and extract text, read aloud, identify people, currency, barcodes, colors, and detect light. The ORCAM and iPhone can increase the overall FIM score average by approximately (3-4.5) once training is provided, including safety awareness (5.10), product identification (4.5), facial recognition and hand-eye coordination with use of camera scores (4.5). The iPhone and ORCAM technologies can improve the quality of life of older Low Vision Veterans and can measurably improve their independence and with the issuance of a smart phone and specialized training provided.

